# Acid-sensing ion channel 1a modulation of apoptosis in acidosis-related diseases: implications for therapeutic intervention

**DOI:** 10.1038/s41420-023-01624-6

**Published:** 2023-09-04

**Authors:** Zhenyu Zhang, Minnan Chen, Wenjing Zhan, Yuechun Chen, Tongtong Wang, Zhonghua Chen, Yifei Fu, Gang Zhao, Dong Mao, Jingjing Ruan, Feng-Lai Yuan

**Affiliations:** 1 Institute of Integrated Chinese and Western Medicine, Affiliated to Jiangnan University, Wuxi, Jiangsu 214041 China; 2 Nantong First People’s Hospital, Nantong, 226001 China; 3 The Key Laboratory of Anti-Inflammatory and Immune Medicine, Anhui Medical University, Hefei, 230032 China; 4 Orthopaedic Institute, Wuxi 9th People’s Hospital Affiliated to Soochow University, Wuxi, 214062 China; 5 Department of Respiratory and Critical Care Medicine, The First Affiliated Hospital of Anhui Medical University, Hefei, 230022 People’s Republic of China

**Keywords:** Molecular biology, Pathogenesis, Apoptosis

## Abstract

Acid-sensing ion channel 1a (ASIC1a), a prominent member of the acid-sensing ion channel (ASIC) superfamily activated by extracellular protons, is ubiquitously expressed throughout the human body, including the nervous system and peripheral tissues. Excessive accumulation of Ca^2+^ ions via ASIC1a activation may occur in the acidified microenvironment of blood or local tissues. ASIC1a-mediated Ca^2+^‑induced apoptosis has been implicated in numerous pathologies, including neurological disorders, cancer, and rheumatoid arthritis. This review summarizes the role of ASIC1a in the modulation of apoptosis via various signaling pathways across different disease states to provide insights for future studies on the underlying mechanisms and development of therapeutic strategies.

## Facts

1. ASIC1a is an ion channel with homotrimer or heterotrimer present in various tissues/cells in the central and peripheral areas.
2. Acidosis-activated ASIC1a regulates cell apoptosis by promoting calcium influx.
3. ASIC1a exhibits variable regulatory effects on apoptotic processes, promoting or inhibiting apoptosis based on the specific disease context.

## Open questions

1. In the pursuit of pharmacological development, what strategies can be used to selectively design ASIC1a blockers capable of modulating apoptotic pathways while preserving the normal physiological functions of the channel?
2. Is there a prospect for artificial ion channels to exert regulatory influence over apoptotic pathways?
3. In the context of tissue biology, how does ASIC1a achieve a delicate equilibrium in modulating the apoptotic, proliferative, and autophagic processes?

## Introduction

Acidosis, a pathological condition in which the pH is significantly dropped with a rise in the acidity of the blood and other body tissues, can result from pathological conditions, including tissue inflammation, ischemic stroke, traumatic brain injury, and epileptic seizures[1–4]. The presence of extracellular acid causes the rapid activation of proton-sensing ionotropic receptors[5, 6]. These channels mainly facilitate acid transduction, which allows cations to enter the cell and transform an acidic extracellular environment into a biological signaling event. Acid-sensing ion channels (ASICs), transient receptor potential vanilloid 1, and a group of proton-sensing G-protein receptors, including ovarian cancer G-protein-coupled receptor 1 (also known as GPR68), are examples of proton-sensing ion receptors[7–9]. ASICs involved in acid sensing have attracted significant interest as class of receptors.

**Fig. 1:**
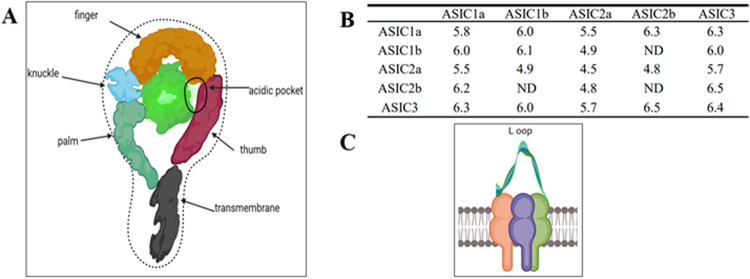
ASIC1a structure diagram, cross-sectional view, and the activation pH of ASICs. **A** Schematic diagram of the structure of ASIC1a. ASIC1a has a fist-like structure, including fingers, palm, thumb, knuckle, acidic pocket, transmembrane, and other structures. **B** Sectional view of ASIC1a. ASIC1a is composed of three subunits and contains a loop. **C** Activation pH of some homopolymers or heteromers of the ASIC family.

The ENaC/DEG ion channel superfamily includes cation channels gated by protons, such as ASICs[10]. Nearly 500 amino acids make up the ASIC subunit, which has two hydrophobic transmembrane (TM) domains, namely a large extracellular loop rich in cysteine and intracellular N- and C-termini [11–13]. The ASIC channel proteins consist of three distinct complexes composed of homomeric and heterotrimeric ASIC subunits (Fig. 1A, C) [11–16]. Acid-sensing ion channel 1a (ASIC1a), acid-sensing ion channel 1b (ASIC1b2), acid-sensing ion channel 2a (ASIC2a), acid-sensing ion channel 2b (ASIC2b), acid-sensing ion channel 3 (ASIC3), and acid-sensing ion channel 4 (ASIC4a) are the four unique ASICs [17–20].

The exclusive permeability of ASIC1a to Ca^2+^ among all ASIC subtypes and its involvement in acidosis have been extensively investigated [21–24]. Previous studies have established the abnormal expression of ASIC1a in diverse pathogenic processes and its role in promoting disease progression by regulating apoptosis, a fundamental cellular process that is either a normal physiological activity or an aberrant pathogenic behavior [25]. Several diseases are characterized by dysregulated apoptotic pathways, underscoring the paramount importance of investigating the intricate regulation of apoptosis [26–29]. However, a comprehensive summary and categorization of the distinct mechanisms by which ASIC1a regulates apoptosis in neurological disorders, cancer, and rheumatoid arthritis (RA) is lacking. To address this gap in knowledge, this review summarizes the role and mechanism of ASIC1a in the regulation of apoptosis.

## ASIC1a

### Overview of ASIC1a

#### Structure

The architecture of each ASIC1 subunit is characterized by a domain arrangement resembling an upright forearm and clenched hand [15]. Notably, residues from the palm, ball, finger, and thumb domains combine to generate an acidic pocket in the extracellular domain, which is extremely negatively charged. Crucially, two pairs of carboxyl-carboxylate interactions that take place unusually close together between the side chains of aspartate or glutamate residues allow the molecule to sense pH [30–32].

The central palm domain of each ASIC1 subunit is made up of a 7-strand sheet, with strands 1 and 12 attaching to the TM1 and TM2 domains and strands 9 and 10 linking to the thumb domain [15, 33]. The knuckle and finger domains are located above the palm domain, with the former including many helices as well as non- and non-structures and the latter including multiple short helices (6 and 7). Disulfide linkages found in abundance in the thumb domain interact with the fingertip domain. The little five-strand ball domain, bordered by the palm, knuckle, finger, and thumb domains, is situated in the middle of the “clenched hand” [15, 33]. Furthermore, Glu-79 and Glu-416, which are essential for proton gating and desensitization, are found in the lower palm domain [15]. The 9–4 loop of the thumb undergoes displacement after proton binding in the extracellular domain, which causes the TM1 domain to rotate and stabilize the channel in the closed state [16].

The TM domain of ASIC1 has a structural arrangement resembling an hourglass, with each TM domain consisting of two elongated helices. The three-fold axis of crystallographic symmetry connects the three subunits that makeup TM1 and TM2 symmetrically [17, 18]. The putative ion channel pore is formed by the TM2 helices, whereas most inter-helical interactions in TM1 take place inside the lipid bilayer [9, 17]. An ion-selective pore is created by the arrangement of the six TM helices. The cation selectivity of ASIC1 is influenced by the net negative electrostatic potential of the intracellular environment. Upon exposure to extracellular acid, the channels undergo rapid activation. The ASIC1a pore, once unmasked, primarily supports an excitatory inward current carried by Na^+^ and K^+^ [34–36]. Notably, homomeric assemblies of ASIC1a and human ASIC1b, as well as heteromeric ASIC1a/2b complexes, exhibit minimal Ca^2+^ permeability [36].

#### Electrophysiology

ASICs exhibit three distinct conformational states, namely closed (resting), open (conducting), and desensitized (proton-bound). Under normal physiological conditions, these channels are predominantly in a closed and resting state [37–39].

Depending on the makeup of their subunits, different ASICs can be differently affected by variations in extracellular pH. With an activation threshold close to pH 7.0 and a pH_50_ (half maximal activation) of approximately 6.2, ASIC1a is especially sensitive to protons [40–43].

ASIC3 demonstrates a transient component with a pH_50_ of ~6.2 and a sustained component with a pH_50_ of ~4.3 [44–46]. ASIC1b, much like ASIC1a, exhibits a pH_50_ of ~6.0 [47–49]. In contrast, the least sensitive to acidic pH is ASIC2a, with a pH_50_ of around 4.4 [35, 36]. Homomeric ASIC2b and ASIC4 channels do not react to proton stimulation [37, 38], and endogenous ligands for these receptors have yet to be identified [39, 40].

**Fig. 2:**
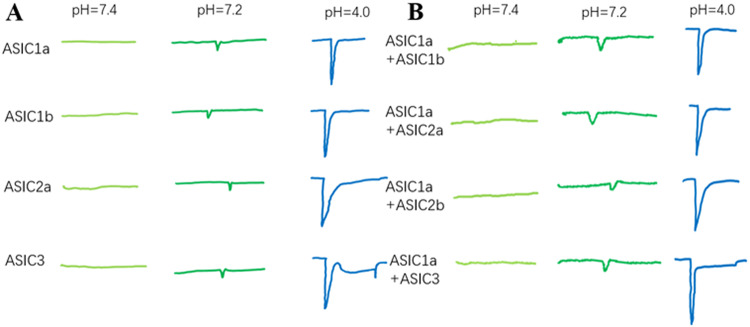
Schematic diagram of ASIC current induced by cells in Na-Ringer using test solutions of different pH values. **A** Acid-induced current of homogenous ASICs. Shown in the figure is a schematic diagram of the current traces, indicating the response of homogeneous ASIC subunits to low pH values. **B** Schematic diagram of current traces at different pH values after ASIC1a is combined with another ASIC subunit, indicating that the heterogeneous ASIC subunit responds to low pH values. Note: This figure is a schematic diagram.

ASICs can attain steady-state desensitization and transition from a closed to a desensitized state without undergoing the activation process (Fig. 1B and 2). In instances where there is an insignificant increase in the proton concentration in the environment, the channels are not activated but respond to subsequent potent acid stimuli, which may be weaker or nonexistent [50]. ASIC1a desensitization begins at pH levels marginally below 7.4, with sustained acidification leading to the complete desensitization of all channels at pH values below 7.1. Consequently, ASIC1a is unable to encode continuous acidity owing to complete desensitization, although it can recover from desensitization within a few seconds. Notably, ASIC1a may undergo desensitization without apparent activation; its semi-desensitized pH maximum is at 7.15 (Fig. 2).

#### Distribution

The distribution and expression range of ASIC1a in various tissues in vivo determine the significance of this ion channel family [51]. ASIC1a is present in diverse mammalian tissues, including those of the nervous system [52–56], bone [57], synovial tissues, intervertebral discs (IVDs), intestinal and bladder epithelial cells [58–60], and smooth muscles [16, 61]. By maintaining the extracellular pH at 7.4, ASIC1a serves various physiological functions [18]. It participates in sensory perception, nociception, mechanosensation, taste transduction, synaptic plasticity, learning and memory, fear conditioning, retinal physiology, and cardiovascular homeostasis [62]. Additionally, ASIC1a expression is upregulated in various brain cancers, implying that it has a role in tumor physiology [63, 64].

### ASIC1a in the regulation of apoptosis machinery

#### ASIC1a in hypoxic-ischemic conditions

ASIC1a plays a substantial role in the etiology of illnesses connected to ischemic damage, such as retinal ischemia and strokes. In the visual pathway, retinal ganglion cells (RGCs) are essential; their death by apoptosis causes progressive vision loss, with retinal ischemia acting as a potentially blinding mechanism underpinning a number of sight-threatening diseases. Prior studies have detected *ASIC1* mRNA in rabbit [65], mouse [66], and rat [67] retinae.

The high expression of ASIC1a channels in rat RGC primary cultures and retinal slices suggests that these channels are crucial in the ischemia-induced cell death of RGCs. In cultured RGCs, the expression and functionality of ASIC1a channels were upregulated following hypoxia. Ratiometric Ca^2+^ imaging data showed that the (Ca^2+^) ion concentration of RGCs increased in response to a decrease in pH, which emphasizes the crucial function of ASIC1a channels in RGCs. The inhibitors amiloride and psalmotoxin 1 can acutely block the ASIC1a channels, significantly reducing RGC death in vitro [68].

In the retina, ASIC1a upregulation induced an influx of extracellular calcium, which may activate calcium-sensitive calpain-1. Similarly, ASIC1a contributes to the mechanism by which renal tubular epithelial cell apoptosis brought on by ischemia/reperfusion (I/R) occurs in the kidney. Renal I/R increases ASIC1a expression both in vivo and in vitro, confirming that ASIC1a is expressed in the kidney. ASIC1a-specific inhibitor psalmotoxin-1 (PcTx1) suppressed H/R-induced apoptosis, especially early apoptosis, in a dose-dependent manner [69].

The I/R model induced acidification in the local microenvironment and ASIC1a activation, resulting in extracellular proton accumulation and Ca^2+^ influx, leading to a loss of mitochondrial membrane potential, an increase in cleaved caspase-3, and the apoptosis of renal tubular epithelial cells.

The mechanisms by which ASIC1a contributes to renal I/R injury are linked to its calcium permeability. PcTx1, a specific inhibitor of ASIC1a, has the potential to treat acute kidney damage by reducing ischemic renal injury via ASIC1a inhibition [69, 70].

#### ASIC1a regulates neuronal apoptosis

**Fig. 3:**
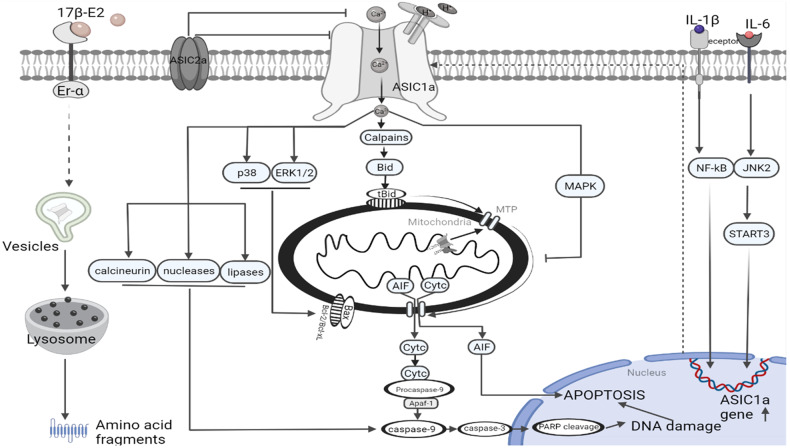
Roles of ASIC1a in apoptosis signal pathways. The white background on the left demonstrates the inhibition of apoptosis by inhibiting ASIC1a or promoting the degradation of ASIC1a, which may involve 17β-E2 and ASIC2a. The pink background on the right summarizes the promotion of the occurrence and development of apoptosis by activating or increasing ASIC1a, which may involve hydrogen ions, calcium ions, IL-1β, and IL-6.

In various neurological diseases, ASIC1a acts as a proton receptor to mediate acidosis-induced neuronal damage. In ischemic brains, glycolysis produces lactic acid as a byproduct, which, in combination with protons generated by ATP hydrolysis, reduces the pH to 6.0 [71, 72]. Acidosis is a major contributor to neurological damage. Empirical evidence supports the notion that a modest and physiologically relevant reduction in extracellular pH triggers the activation of ASIC1a, which subsequently results in direct increases in Na^+^ and Ca^2+^ levels within the cytosol and mitochondria via ASIC1a-mediated processes [73] (Fig. 3).

In the past, glutamate receptors have been linked to excess Ca^2+^ in the ischemic brain. However, clinical studies using glutamate antagonists to protect the brain from Ca^2+^ toxicity did not produce encouraging results. The activation of Ca^2+^ signals in ASIC1a-overexpressing HEK293 T cells persists despite the absence of voltage-gated calcium channels, which provides evidence for an ASIC1a-dependent Ca^2+^ response [74, 75] (Fig. 3).

ASICs are inhibited by amiloride, whereas homomeric ASIC1a channels are specifically blocked by pcTx1. Both inhibitors can shield neurons from damage caused by acid exposure and oxygen-glucose deprivation, according to in vitro studies [76]. In addition, the injection of these inhibitors decreased infarct volume in animal models of middle cerebral artery occlusion (MCAO). In MCAO rat models, the Chinese herbal supplement sophocarpine demonstrated neuroprotective potential, which can presumably be explained by its anti-ASIC1 and anti-apoptotic characteristics [77]. Furthermore, MCAO-induced neuronal injury was not fatal in ASIC1a knockout mice, emphasizing the importance of ASIC1a activation in the pathophysiology of brain ischemia/stroke [76]. As a result, Ca^2+^-permeable ASIC1a channel activation during brain ischemia can result in neuronal damage, and ASIC1a inhibition is a prospective therapeutic target for neuroprotection in this situation [18].

Following cerebral ischemia, an intrinsic apoptotic signaling cascade is initiated. Cerebral ischemia activates ASICs, increasing calcium levels in the cytosol. Following the activation of calpains, increased intracellular calcium levels cleave Bid into truncated Bid (tBid) fragments. Proapoptotic proteins, like Bad and Bcl-2-associated X protein (Bax), are generally neutralized by antiapoptotic B-cell leukemia/lymphoma 2 (Bcl-2) family members, like Bcl-2 and Bcl-2-like protein 1 (Bcl-xL), in the mitochondrial membrane, where they interact with tBid. Cytochrome c (Cyt-c) or apoptosis-inducing factor (AIF) is released when mitochondrial transition pores open as a result of tBid heterodimerization with proapoptotic proteins. In the cytoplasm, Cyt-c then joins with procaspase-9 and apoptotic protein-activating factor-1 to form an “apoptosome,” which in turn activates caspase-9 and subsequently caspase-3. When caspase-3 is activated, it cleaves nuclear DNA repair enzymes, such as poly(ADP-ribose) polymerase (PARP), which causes DNA damage and cell death. AIF moves into the nucleus and, in the absence of caspase activation, causes severe DNA breakage and cell death (Fig. 3).

Wang et al. identified a new pattern of subcellular localization and intracellular function for ASIC1a. The study revealed that ASIC1a is present in the mitochondria of mouse cortical neurons, where it physically interacts with adenine nucleotide translocase, a protein of the mitochondrial inner membrane that has been theorized by some scientists to be a part of the mitochondrial permeability transition (MPT) pore, although this assertion has been disputed [78]. MPT pores, a factor in oxidative neuronal cell death, may be regulated or composed of mitochondrial ASIC1a [79, 80]. MPT activity influences the ability of mitochondria to uptake Ca^2+^ ions [81]. The Ca^2+^ retention capacity and mitochondrial Ca^2+^ uptake rate of brain mitochondria isolated from ASIC1a-/- mice are significantly increased [78, 82]. In comparison to those of wild-type mice, neurons of ASIC1a-null mice show increased resistance to H_2_O_2_-induced Cyt-c release and inner mitochondrial membrane depolarization [78]. ASIC1a-/- neurons exhibit a reduction in MPT-dependent H_2_O_2_-induced neuronal death. According to these findings, mitochondrial ASIC1a may have a novel role in controlling MPT-dependent neuronal death, a crucial stage in brain injury brought on by ischemic stroke and neurodegenerative diseases. Azoulay et al. hypothesized that mtASIC1a functions as a channel that controls mitochondrial Na+ input [82]. The mitochondrial Na^+^ signal induced by pH 7.0 is mediated by channels sensitive to PcTx1 in both permeabilized HEK293T cells overexpressing ASIC1a and primary cortical neurons, suggesting that these Na^+^ signals are mediated, at least in part, by mtASIC1a [82]. As a result, mtASIC1a may directly control metabolic activity and mitochondrial membrane potential [83]. Although speculative, plasma membrane ASIC1a (death initiator) and mtASIC1a (death executor) may control ischemia cell death at several stages [84] (Fig. 3).

ASIC1a is involved in neuronal apoptosis triggered by insulin deprivation. Insulin deprivation can induce apoptosis in NS20y cells, while the administration of PcTx-1 can reverse this effect [85]. Endoplasmic reticulum stress (ERS), caused by low insulin levels, increases the expression of CHOP and glucose-regulated protein 78 (GRP78) in the ERS group compared with the control group. CHOP translocates to the nucleus and improves its binding to C/EBP in the process of insulin deficiency-induced nerve cell apoptosis, lowering the negative regulatory effect of C/EBP on ASIC1a and raising the level of nerve cell apoptosis [86] (Fig. 3).

#### ASIC1a regulates apoptosis in cancer

Cancer is a neoplastic disorder characterized by aberrant angiogenesis, hypoxia, and acidosis within the tumor microenvironment [87–89]. The growth, invasion, and metastasis of cancerous cells are promoted by acidification of the extracellular matrix [90, 91]. ASIC1a, a common acid-sensing receptor on the cell surface and a member of the DEG/ENaC family of ASICs, is important in the pathogenesis of many cancers, including gliomas, lung cancer, liver cancer, pancreatic cancer, breast cancer, and hepatocellular carcinoma. In numerous tumor types, ASIC1a significantly modulates the degree of malignancy and exacerbates tumor progression by regulating the apoptotic signaling of neoplastic cells [84, 92–94]. Alteration in the tumor microenvironment correspondingly shifts the functional significance of ASIC1a. ASIC1a assumes a pro-apoptotic role across diverse cell types, including neuronal cells, although manifests as an anti-apoptotic mediator within the tumor microenvironment[95–98].

Gliomas are aggressive and highly invasive. They originate from glial progenitor cells in the central nervous system [99, 100]. In patients with glioma, the expression of *ASIC1* mRNA is upregulated, while the expression of *ASIC2* and *ASIC3* is downregulated compared with levels in healthy individuals. ASIC1a subunit-containing channels may be involved in various processes in malignant cells, as they are expressed in U251 MG and A172 glioma cells but not in normal astrocytes [83]. ASIC1a mediates the inward movement of amiloride-sensitive cations into advanced glioma cells [83]. Mambalgin-2, an ASIC1a-specific inhibitor, induces cell cycle arrest and apoptosis in glioma cells by interacting with the ASIC1a channel. The inhibitor binds to the ASIC1a channel in the upper half of the thumb domain (residues Asp-349 and Ph-350) to interfere with the palm and beta sphere domains of the nearby subunit, inhibiting cyclin D1 and CDK phosphorylation, inducing apoptosis, and keeping the channel closed regardless of the pH [101]. These results indicate that ASIC1a promotes glioma occurrence and development by inhibiting apoptosis in U251 MG and A172 glioma cells. Additionally, Ma et al. revealed that extracellular acidification induces apoptosis in C6 glioma cells via ASIC1a. Apoptotic cell percentages increase with decreasing pH, while ASIC1a knockout reduces extracellular acid-induced apoptosis. This process may be accociated with calcium influx in C6 cells [102] (Fig. 3).

Melanoma, a malignant neoplasm, is known for its aggressive behavior. The proliferation, motility, and invasion of primary metastatic melanoma cells are considerably enhanced by the acidification of extracellular mediators [103, 104]. According to Lyukmanova et al., metastatic melanoma cells express more acid-sensitive channels with the subunits ASIC1a, -ENaC, and -ENaC than corresponding estimates in healthy keratinocytes. Additionally, administering a recombinant analog of mambalgin-2 effectively inhibits the growth, migration, and invasion of acidification-induced metastatic melanoma cells, promotes cell apoptosis, inhibits the expression of the proto-oncogenes *CD44* and *Frizzled-4*, and lowers the phosphorylation of the transcription factor SNAI. These results offer insights into therapeutic targets to control metastatic melanoma [105].

Non-small cell lung cancer, which includes lung adenocarcinoma, accounts for approximately 85% of all lung cancer diagnoses and is a highly frequent malignancy globally [106, 107]. Mambalgin-2 effectively suppresses the development and migration of A549, metastatic Lewis P29 cells, and WI-38 cells but has no similar effect on normal fibroblasts. Mambalgin-2 interacts with ASIC1a, -ENaC, and -ENaC iso-tramer channels, causing cell cycle arrest in the G2/M phase, promoting cell death, and preventing the spread and development of lung cancer, as determined by affinity chromatography on lung adenocarcinoma cells.

The development of novel therapeutic approaches for the treatment of non-small cell lung cancer, in particular lung adenocarcinoma, is significantly impacted by these findings [108].

Aside from glioma and lung cancer, the abnormal expression of ASIC1a has also been observed in liver cancer, a prevalent malignancy that has been identified as the primary cause of cancer-related death globally [76, 77]. ASIC1a facilitates the migration and invasion of liver cancer cells. Acidification of the tumor microenvironment results in H^+^ accumulation, leading to H^+^ competition for Ca^2+^ binding sites on ASIC1a, cell membrane depolarization, β-catenin phosphorylation, and ubiquitination, increased Ca^2+^ influx, and, ultimately, apoptotic resistance of hepatoma cells. ASIC1a deletion, conversely, causes G1/S phase block and cell death and prevents liver cancer cells from proliferating. The results suggest ASIC1a as a potential new therapeutic target for the treatment of liver cancer [109] (Fig. 3).

#### ASIC1a regulates chondrocyte apoptosis in RA

RA, a chronic autoimmune condition, is characterized by escalating joint inflammation. The affected joints exhibit symmetrical synovial inflammation, resulting in cartilage degradation, bone erosion, and functional impairment [78, 110]. A possible factor contributing to the pathogenesis and development of RA is extracellular acidification [111].

Particularly, in patients with active RA, the pH of synovial fluid may drop below 6.0. Additionally, ASIC1a expression levels are higher in patients with RA and rat models of RA than in normal tissues, suggesting that ASIC1a is involved in the pathogenesis of RA [112].

ASIC1a is an essential component in the transmission of apoptotic signaling molecules in articular chondrocytes. The inhibition of ASIC1a with prevents acid-induced chondrocyte apoptosis [111]. The ASIC1a subunit is activated by extracellular acidosis and mediates Ca^2+^ overload, resulting in acid-induced rat articular chondrocyte damage [82]. According to Yuan et al., articular chondrocytes cultivated under an extracellular pH of 6.0 had elevated intracellular calcium levels. The increase in Ca^2+^ and acid-induced chondrocyte damage in articular chondrocytes was decreased by amiloride. The inhibitory effect of amiloride on ASIC1a mitigated articular cartilage degradation and higher levels type II collagen and aggrecan mRNA and protein expression in rats with adjuvant arthritis (AA) [86]. Excess calcium in the cytoplasm has been found to cause apoptosis by various mechanisms. Hu et al. demonstrated the involvement of ASIC1a in the transmission of apoptotic signaling molecules in articular chondrocytes. ASIC1a activates calpain and calcineurin through a Ca^2+^ overload. Amiloride inhibits caspase-3 activity and downregulates Ca^2+^-dependent signaling pathways, such as calpain and calcineurin, which protect articular chondrocytes from acid-induced apoptosis [113]. Apoptosis in acid-induced chondrocytes was reduced by amiloride treatment in a dose-dependent manner; following extracellular acidification, 54.4% of chondrocytes died. ASIC1a controls the expression of several Bcl-2 proteins, such as the anti-apoptotic BCL-2 and Bcl-xL as well as the pro-apoptotic Bax and BID. Amiloride affects the mRNA expression of apoptosis-related Bcl-2 family genes and caspase-3/9 activity, which in turn partially restores mitochondrial membrane potential in chondrocytes produced by extracellular acid [114] (Fig. 3).

Interleukin-1 beta (IL-1), tumor necrosis factor alpha (TNF-), and interleukin-6 (IL-6) have elevated pro-apoptotic effects in acid-stimulated articular chondrocytes, and this was partially ascribed to their role in controlling ASIC1a expression and activity. Zhou has shown that IL-1, TNF-, and IL-6 therapy elevated ASIC1a levels in a time- and dose-dependent manner. The signaling pathways for Janus kinase 2 and signal transducer and activator of transcription 3 (JAK2/STAT3) as well as mitogen-activated protein kinase/nuclear factor-kappa B (MAPK/NF-B) may be partially responsible for this rise. TNF and IL-1 increased NF-κB binding to the ASIC1a promoter, increasing ASIC1a activity. Pyrrolidine dithiocarbamate abolished this effect. Additionally, IL-1 and TNF pretreatment significantly raised levels of intracellular Ca^2+^, cleaved PARP, caspase-3, and caspase-9 and resulted in the loss of mitochondrial membrane potential (m) in acid-induced articular chondrocytes. By using PcTx1 to block ASIC1a channels, these effects might be partially reverted [115, 116]. Additionally, ASIC2a has a stronger inhibitory effect against acid-induced articular chondrocyte apoptosis in the presence of PcTx1 and Apamin-Sensitive Potassium Channel Blocker 2 (APETx2). By reducing intracellular Ca^2+^ levels and blocking the p38 and extracellular signal-regulated kinase 1/2 (ERK1/2)/MAPK signaling pathways, the overexpression of ASIC2a may exert a stronger protective effect. ASIC2a overexpression increased the levels of *Bcl-2* and *Bcl-xL* mRNA, while decreasing the expression of *Bax* mRNA in acid-stimulated chondrocytes [117] (Fig. 3).

ASIC1a can simultaneously promote autophagy and death in articular chondrocytes activated by acid. Dai et al. demonstrated that ASIC1a increases intracellular Ca^2+^ levels in rat articular chondrocytes, which causes acid to activate autophagy. Following pretreatment with the ASIC1a inhibitor PcTx1 and the calcium-chelating compound BAPTA-AM, the levels of the autophagy-related markers LC3-II and Beclin 1 decreased substantially, with subsequent increases in response to extracellular acid at pH 6.0 [118]. Zhou and colleagues analyzed changes in autophagy and apoptosis over time in distinct stages of animal AA. The results imply that reducing apoptosis while increasing autophagy mitigates chondrocyte damage in the joints of rats with AA.

The autophagy inhibitor 3-methyladenine (3MA) increased synovial fluid inflammation and articular chondrocyte mortality in AA mice in vivo, while the autophagy activator rapamycin attenuated these effects [103]. Additionally, autophagy affected the in vitro death of articular chondrocytes induced by acid. Rat articular chondrocytes pretreated with rapamycin exhibited reduced acid-induced apoptosis, whereas pretreatment with 3MA exacerbated apoptosis. Furthermore, 3MA decreased Beclin 1 expression, prevented the formation of autophagic vesicles, and reversed the conversion of LC3-I to LC3-II. In contrast to a pH 6.0 group, it also markedly boosted the expression of apoptosis-related proteins, like cleaved PARP, caspase-3, and caspase-9, and raised apoptosis rates from 13.90% to 21.54%.

To prevent acid-induced apoptosis in chondrocytes, 17-E2 induces the degradation of ASIC1a through the endoplasmic reticulum (ER) receptors. Exposure to 17-E2 reduced ASIC1a protein levels and associated Ca^2+^ influx in a time-dependent manner. Further research revealed that 17-E2 can promote ASIC1a protein degradation by activating chondrocyte autophagy via the autophagy-lysosomal pathway. In ovariectomized rats with AA, Hang et al. demonstrated that estradiol can protect articular cartilage in vivo by lowering ASIC1a expression, autophagy, and pro-inflammatory markers.

This potential mechanism may be explained by the inhibitory effects of estrogen on ASIC1a expression via interactions with G-protein-coupled estradiol receptor 1 and activation of the PI3K-AKT-mTOR signaling pathway [90] (Fig. 3).

#### ASIC1a regulates IVD apoptosis

IVDs comprise three main cellular components: endplate (EP) chondrocytes in the cephalic and caudal EPs, nucleus pulposus (NP) cells in the inner region, and annulus fibrosus (AF) cells in the outer region [91]. Physiologically, the pH of normal NP ranges between 7.0 and 7.25. However, pathologically degenerated NP tissue removed post surgery exhibits significantly reduced pH values of 5.5–5.6 [108, 119, 120]. The microenvironment of deteriorated IVDs in particular exhibits unique and extreme chemical characteristics, such as an acidic matrix, increased extracellular osmolarity, and impaired feeding. The deteriorated IVD exhibits matrix acidity, which may compromise cell viability and function [121].

The IVD of healthy human participants expresses all four ASIC mRNA species, including *ASIC1, ASIC2, ASIC3*, and *ASIC4* [103, 122]. Notably, these mRNA species are markedly upregulated in IVD after degeneration. In both the AF and NP of the typical IVD, *ASIC2* expression levels were highest among ASIC mRNA species, followed by *ASIC3, ASIC1*, and *ASIC4*.

In the deteriorated IVD, AF exhibited a substantial rise in the number of cells expressing *ASIC1* and *ASIC4*, whereas NP exhibited considerable increases in the expression of *ASIC1, ASIC2*, and *ASIC3* [123, 124].

Cai et al. investigated primary human NPCs generated from patients with IVD degeneration [125]. At pH of 6.0, 6.5, and 7.0, there was a significant rise in the proportion of apoptotic cells, while PcTx1-treated groups showed a decline in the rate of apoptosis. The expression of apoptosis-related proteins, such as Bax and cleaved caspase-3, increased following acidic incubation, while Bcl-2 expression dropped. PcTx1 treatment, however, increased the expression of Bcl-2, while decreasing the expression of Bax and cleaved caspase-3 [120] (Fig. 3).

Non-steroidal anti-inflammatory drugs are commonly recognized as non-specific inhibitors of ASICs. Sun et al. uncovered their potential to modulate ASIC expression in NPCs. Following treatment with ibuprofen, the expression and current of ASIC1 and ASIC3, which are triggered by acidic stress, decreased significantly. Additionally, degeneration-induced cell death was effectively suppressed [124].

Following exposure to acidic stimuli, ASIC1a contributes to the regulation of ERS and promotes NPC apoptosis. Acid-induced activation of ASIC1a upregulates ERS markers. Moreover, the blockade of ASIC1a activity reduces the levels of GRP78, CHOP, and caspase-12.

GRP78, a vital protein involved in ERS, has been found to function as a key mediator of ASIC1a and acid-induced ERS. CHOP and caspase-12, both essential proteins involved in ERS-induced apoptosis, have also been implicated in this process [126].

EP tissue is a form of hyaline cartilage. Li et al. investigated the EP and determined that the activation of ASIC1a triggers apoptosis in the EP by regulating the rise in intracellular Ca^2+^ concentration and calcium-dependent protease activity. By inhibiting calcium-activated signaling pathways such as BAD dephosphorylation, Cyt-c release, and effector caspase activation, the blockade of ASIC1a with PcTx1 and ASIC1a-siRNA may safeguard EP chondrocytes against acid-induced apoptosis [122].

Bone marrow stromal cells (BMSCs) have attracted research interest owing to their vast potential in regenerative medicine. Stem cell transplantation has been explored as a possible means of preventing or reversing the process of disc degeneration [103]. Using a rabbit model, Cai et al. revealed that the transplantation of BMSCs can enhance the regeneration of damaged discs [104]. However, it is possible that BMSC vitality and biosynthetic activity are not sufficient to meet the needs of severely degenerated discs. The harsh microenvironmental conditions in degenerated discs can have deleterious effects on both native and transplanted cells [120].

ASIC1a expression in murine BMSCs has been documented [125].

Furthermore, ASIC1a expression in rat BMSCs was discovered by Cai et al., and its activation was discovered to cause calcium-dependent BMSC death in circumstances that mimicked the acidic microenvironment of the degenerating IVD [109]. ASIC1a activation has been linked to the increase in intracellular Ca^2+^ induced by acid in BMSCs. The Ca^2+^-dependent activation of calpain and calcineurin may result from this rise in Ca^2+^. Additionally, activated calcineurin can promote pBAD dephosphorylation. The resulting dephosphorylated BAD enhances the release of Cyt-c from mitochondria by increasing mitochondrial permeability. Thus, the mitochondrial apoptosis pathway partially mediates acid-induced BMSC injury (Fig. 3). ASIC1a is essential for apoptosis in a variety of tissues and disorders; Table 1 summarizes its distribution and functions.

**Table 1:** Distribution of ASIC1a and mechanisms underlying its regulatory effects on apoptosis.

category	Tissue/cell	function	Receptor	Effect on apoptosis	Species	References
Neuronal apoptosis	Cortex	Linking extracellular pH stimuli to mitochondrial ion signaling .	Mitochondrial calcium overload	Promote	Mouse	^73^
	Monocytes	Promote RAW 264.7 cells apoptosis	NA	Promote	Human	^57^
	Cortical neuronal	Activated NLRP1 inflammasome	NLRP1	Promote	Rat	^137^
	Cortical neurons	Mitochondrial ASIC1a regulate MPT pores	MTP	Promote	Mouse	^79^
	Nucleus pulposus cells (NPCs)	Decreased Bcl-2 and increased Bax, cleaved caspase-3 and senescence-related proteins	Calcium ion	Promote	Rat	^120^
	Nucleus pulposus cells (NPCs)	Regulate ER stress	ER	Promote	Rat	^126^
Cancer	Gliomas	Induced C6 glioma cells apoptosis	Short hairpin RNA	Inhibition	Human	^102^
	Glioblastoma	Inhibition of the Notch signaling pathway and CD133 and aldehyde dehydrogenase 1	Calcium ion	Promote	Human, rat	^138^
	C6 glioma cells	Induce the increase of Ca^2+^ in the wild-type C6 cells	Calcium ion	Promote	Rat	^139^
	Gliomas	Induce apoptosis in U251 MG and A172 cells	Cyclin-dependent kinases (CDK)	Promote	Human	^83^
	Neuroblastoma	ERS and ASIC1a induce neurological damage	CHOP	Promote	Mouse	^85^
	Renal epithelia cell	Induced Ca overflow, loss of ∆ψm and apoptosis	Mitochondria	Promote	Human	^69^
	Bone marrow mesenchymal stem cells (BMSCs)	Increased mitochondrial permeability and mitochondrial-mediated apoptosis	Mitochondria	Promote	Rat	^108^
Intervertebral discs apoptosis	Intervertebral discs	Trigger Ca(2 + )-dependent proteases activity and signaling	Calcium overload	Promote	Rat, human	^122^
	IVDD	Regulates levels of intercellular reactive oxygen species (ROS)	ROS	Promote	Rat	^140^
Rheumatoid arthritis	Articular chondrocytes	Mediated apoptosis in rat articular chondrocytes	Autophagy	Promote	Rat	^113^
		ASIC2a rat articular chondrocyte apoptosis by regulating ASIC1a	ASIC2a	Promote	Rat	^115^
		IL-1β and TNF-α can cytotoxicity upregulation of ASIC1a	Caspase-3/9	Promote	Rat	^112^
		Enhance apoptosis through JAK2/STAT3 and MAPK/NF-κB signaling pathways	JAK2/STAT3	Promote	Rat	^111^
		NGF promote acid-induced apoptosis of chondrocytes by up-regulate ASIC1a	Caspase-9	Promote	Rat	^128^
		17β-E2 suppressed apoptosis, and restored mitochondrial function	Mitochondria	Promote	Rat	^116^
		Increased Ca^2+^	Calcium ion	Promote	Rat	^82^
		Amiloride inhibit the expression levels of calpain and calcineurin	Calcium ion	Promote	Rat	^141^
		Amiloride increasing anti-apoptotic ability and down-regulation of pro-apoptotic factors	Calcium ion	Promote	Rat	^142^
		3-MA increased apoptosis, rapamycin reduced chondrocyte apoptosis	Autophagy	Promote	Rat	^143^
		Estradiol suppressing ASIC1a expression through GPER1	Autophagy	Promote	Rat	^90^

## Conclusions

Learning, memory, and brain activity are just a few of the physiological activities involving the ASIC family of ion channels [105]. ASICs also have a significant impact on several illnesses, including RA, glioma, stroke, and neuronal apoptosis [43, 90, 106, 107] and are an essential member of the ASIC family, as they accelerate disease development by inducing apoptosis in a variety of cell types [106, 109, 125]. For example, ASIC1a activation can cause extracellular calcium influx and induce apoptosis in RGCs, renal tubular epithelial cells, and neurons under hypoxic or ischemic conditions [68]. ASIC1a also regulates anti-apoptotic pathways in different cancers, including glioma, melanoma, and lung and liver cancers, via homologous or heterologous subunits. Inhibiting ASIC1a using mambalgin-2 can significantly promote cancer cell apoptosis and slow disease progression [127]. In contrast, ASIC1a strongly promotes apoptosis in chondrocytes during RA. IL-1β, TNF-α, and IL-6 activate ASIC1a via various pathways, including calpain or ASIC2, leading to calcium influx and chondrocyte apoptosis [111, 112, 115, 116, 128]. Furthermore, ASIC1a can regulate chondrocyte apoptosis through autophagy [113]. In the acidic microenvironment of degraded discs, EP chondrocytes undergo apoptosis due to increased levels of apoptosis-related proteins promoted by ASIC1a activation [120]. Moreover, ASIC1a activation also leads to calcium-dependent apoptosis in BMSCs [108].

As one of the basic biological processes of cells, apoptosis is related to the balance between apoptotic and anti-apoptotic processes. Simply promoting or inhibiting apoptosis, which has been a focus of research, will lead to various toxic side effects in living organisms [129, 130]. Studies have rarely considered the balance between regulating apoptotic and anti-apoptotic processes. Ion channels are very important barriers to communication between cells and the external environment [131]. The targeted regulation or competitive binding of ion channels is an important approach to regulating cellular processes [132]. Examples include the development of a variety of artificial subnanopores, subnanochannels, and subnanoslits with good ion selectivity and permeability for signal communication and biosensing [133, 134]. In addition to the synthesis of artificial channels, the development of new competitive specific channel blockers that are different from PCTX-1 is another strategy to regulate the balance of apoptosis. Research in this area is focused on a reliable and convenient means to study the apoptotic balance. Recent data on ASIC1a have provided novel insights into its mechanisms and functions in apoptosis across various cell types [135, 136]. Such knowledge can provide a scientific basis for diagnosing and treating multiple diseases. However, further investigations are necessary to explore the expression and roles of ASIC1a in understudied tissues or diseases and to understand the factors underlying differences in its effects depending on the tissue environment. We believe that advancements in science, technology, and experimental techniques will bring both opportunities and challenges in ASIC1a research.

### Supplementary information

Submission and informed consent of co-authors
