# Supplementary figures and images for: Acid-sensing ion channel 1a modulation of apoptosis in acidosis-related diseases: implications for therapeutic intervention
Source: Cell Death Discov. 2023 Sep 4;9:330. doi: 10.1038/s41420-023-01624-6 (PMC10477349; doi:10.1038/s41420-023-01624-6)

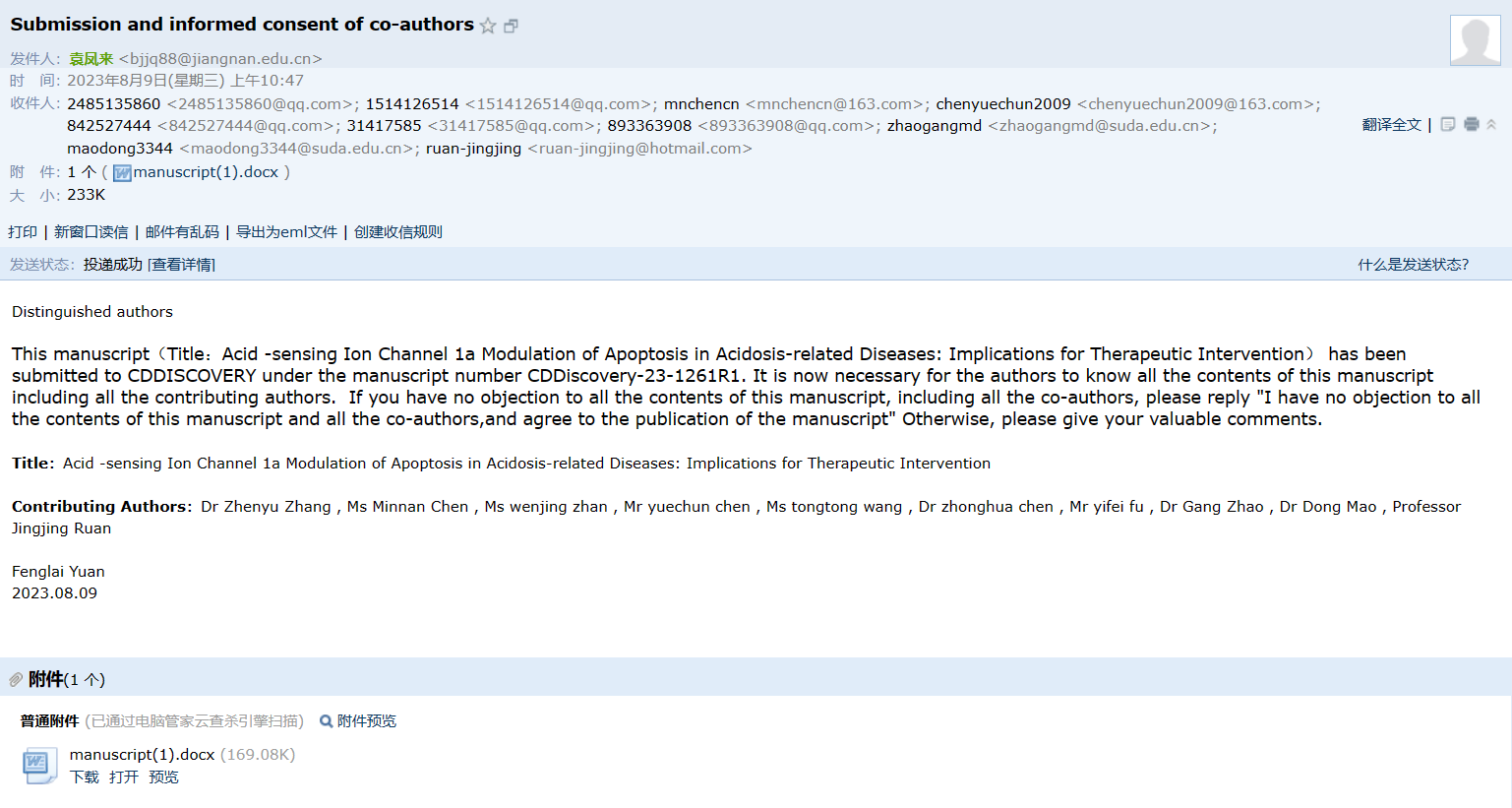

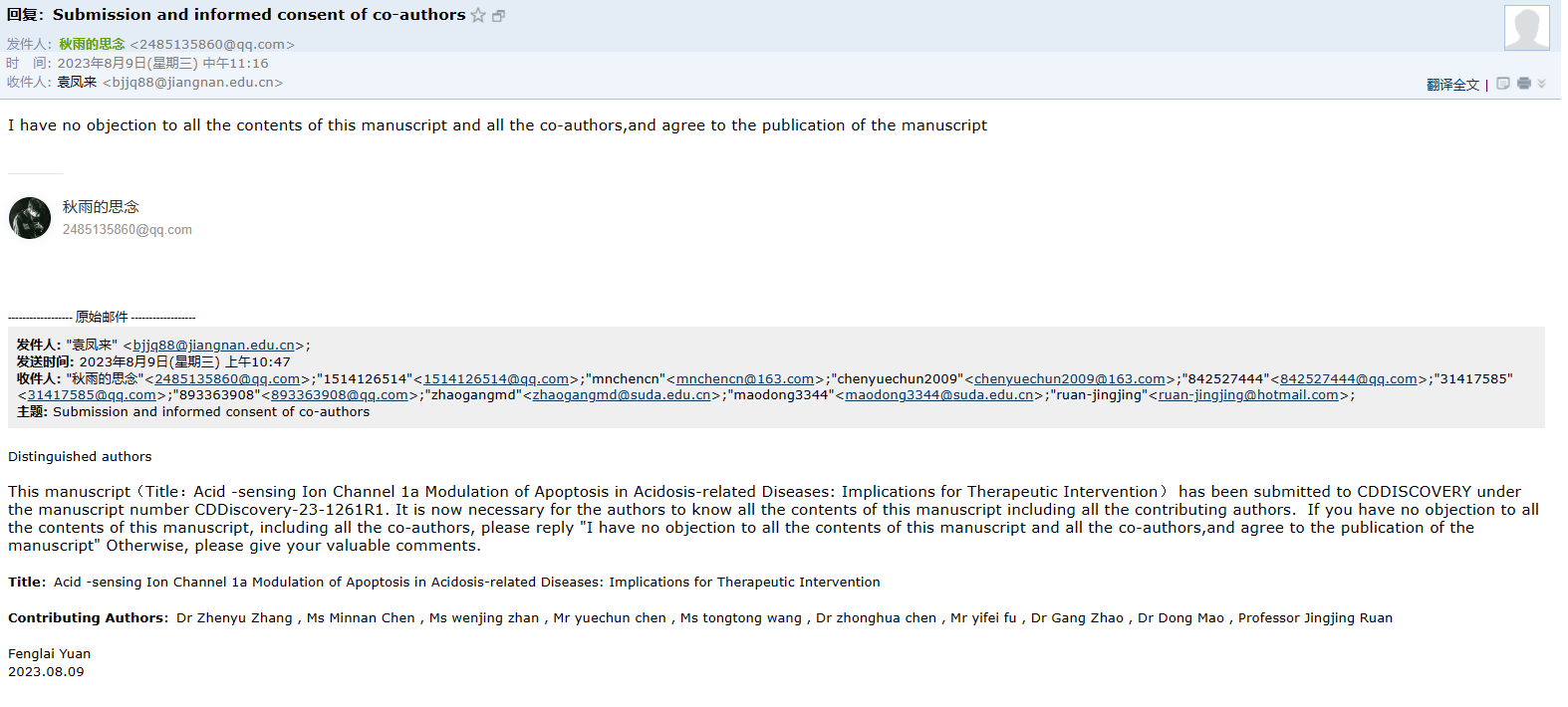

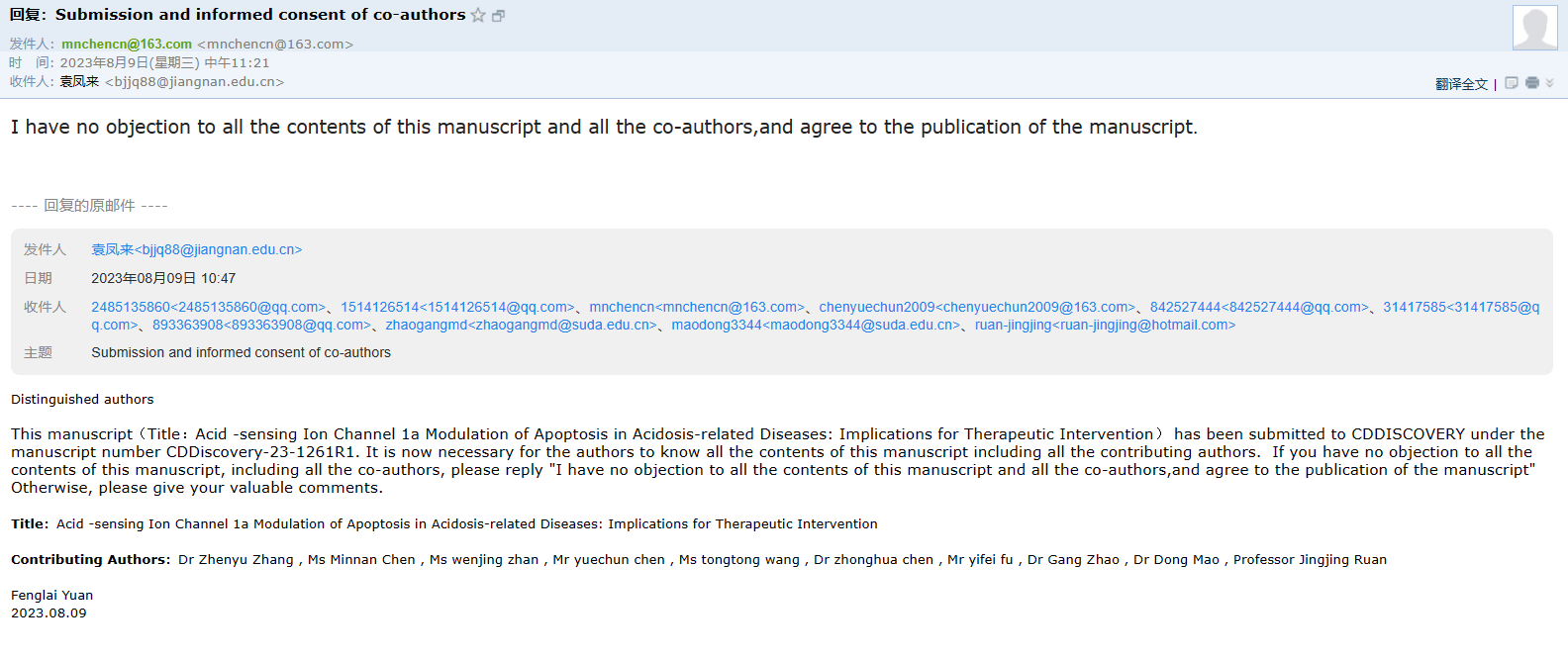

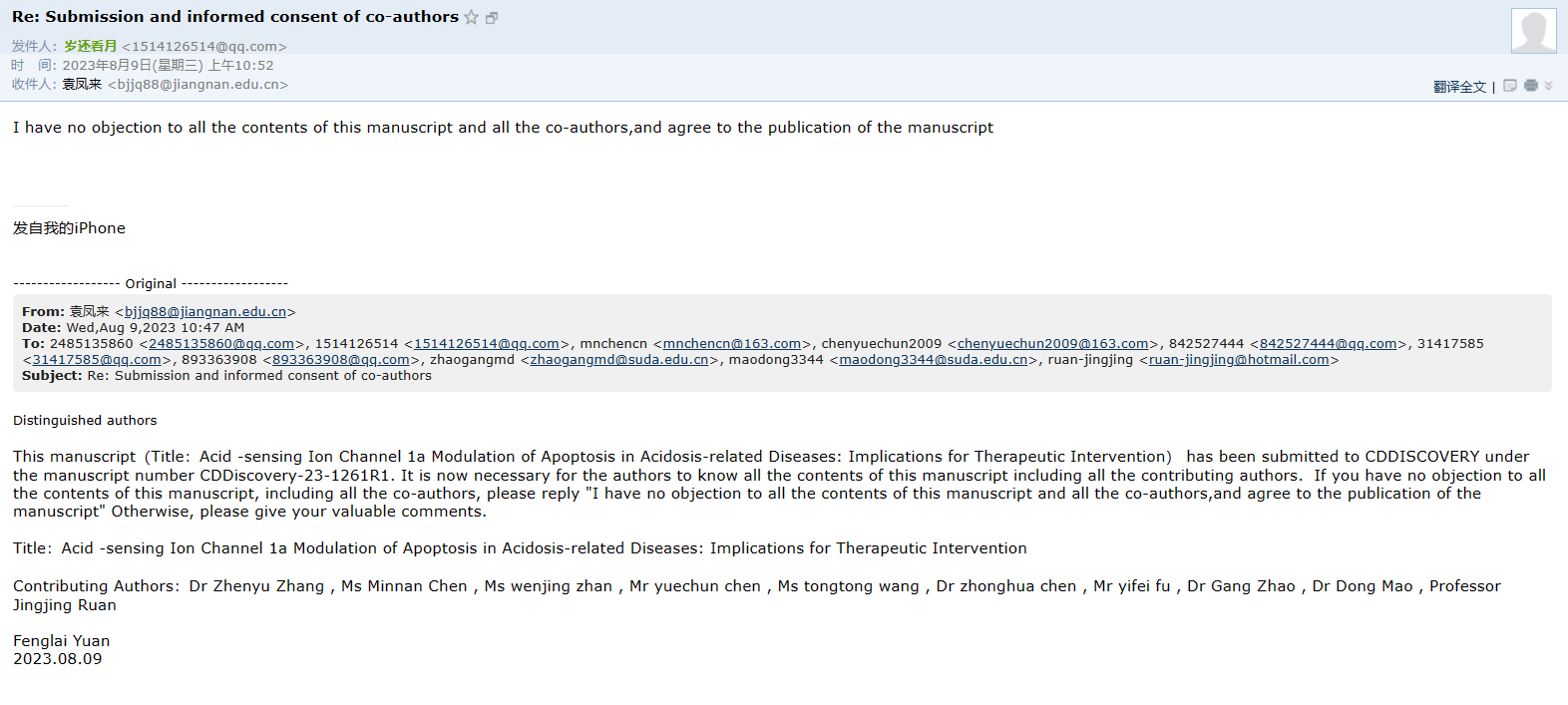

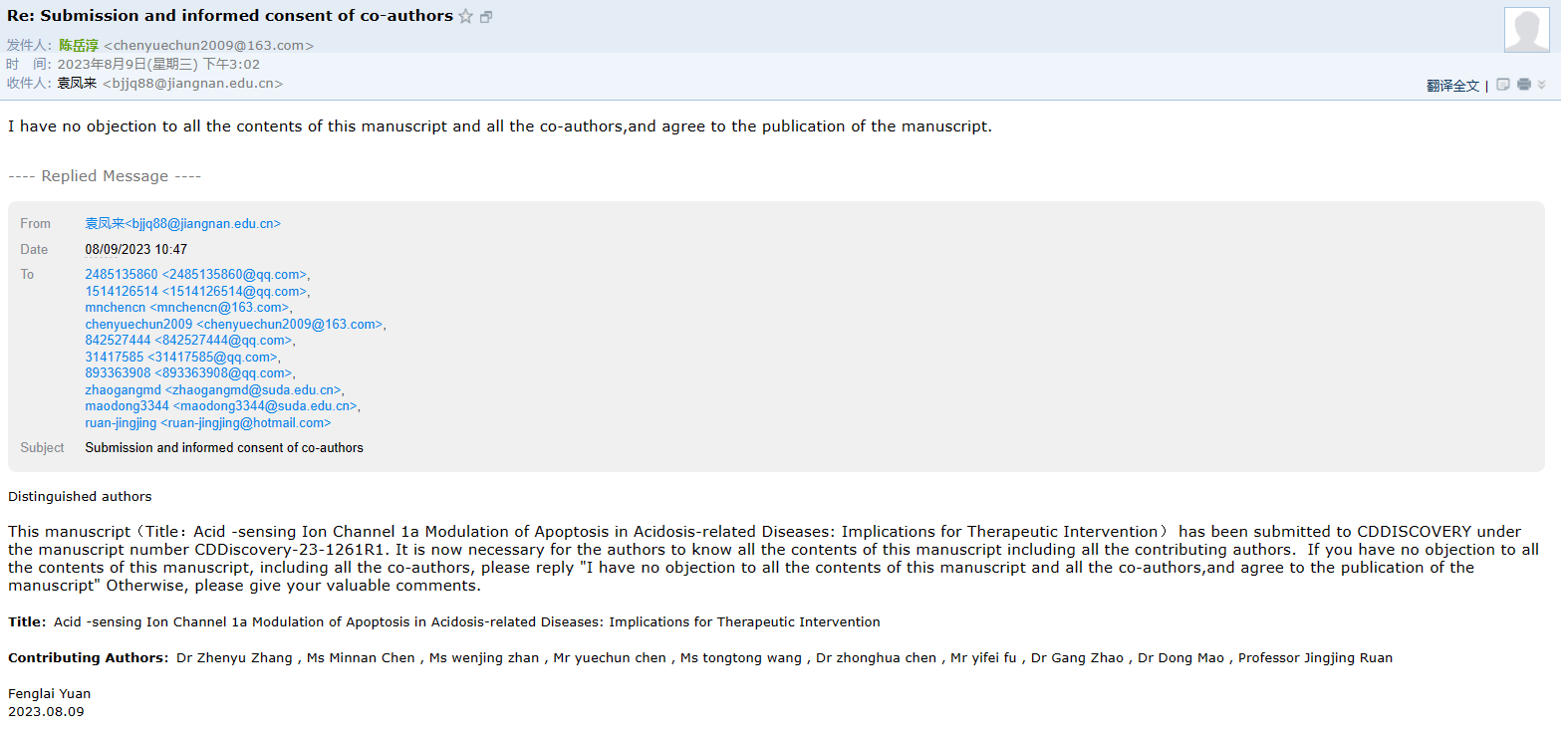

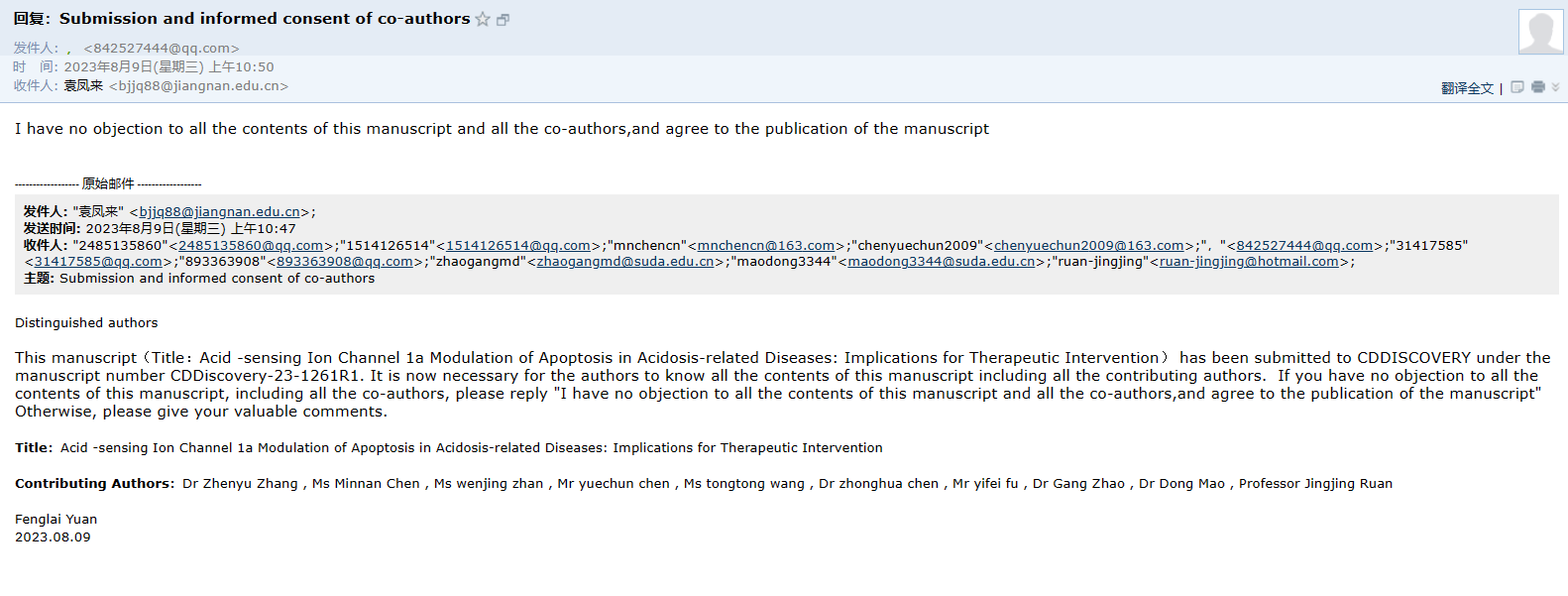

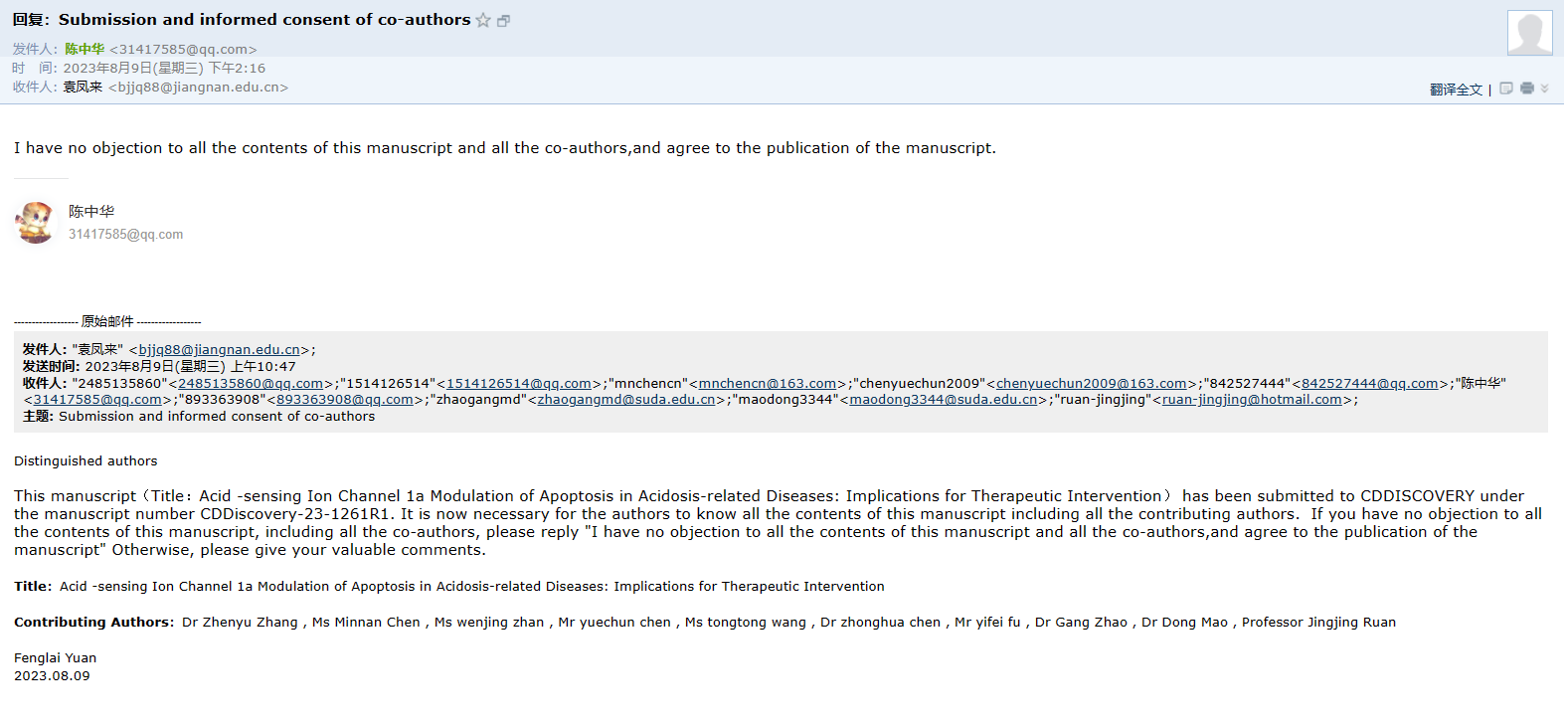

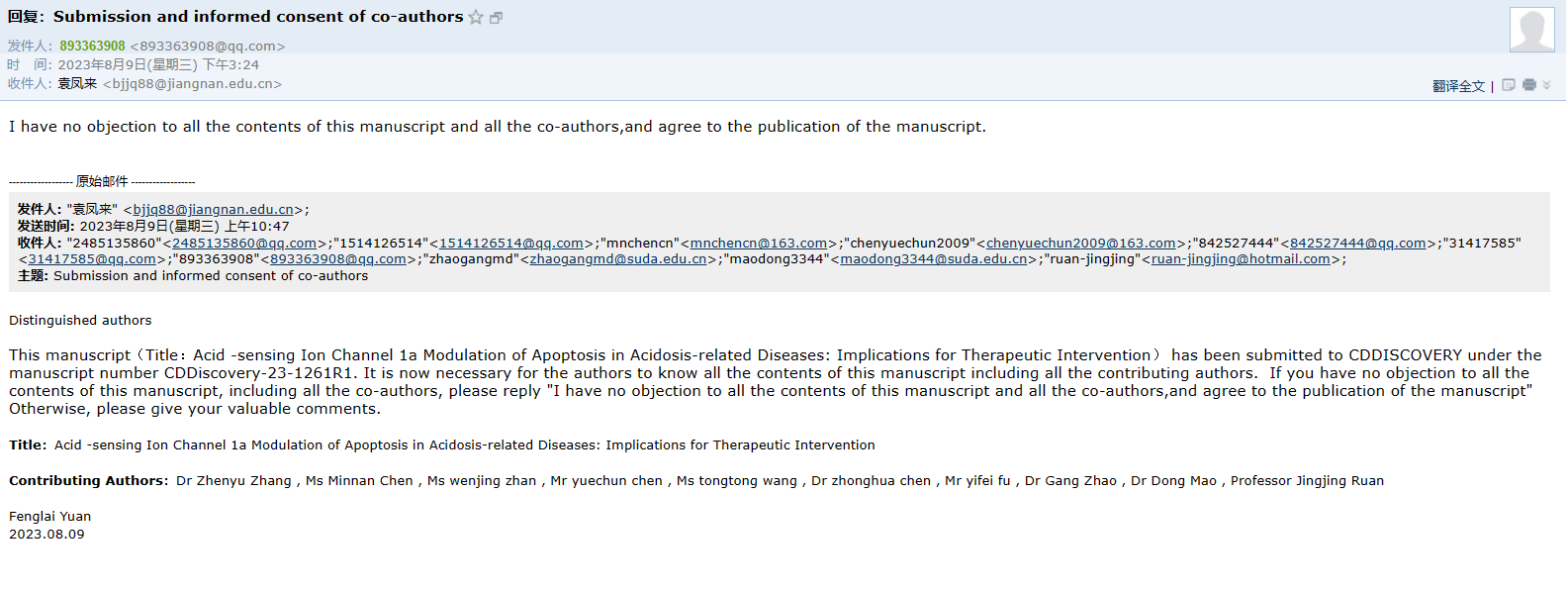

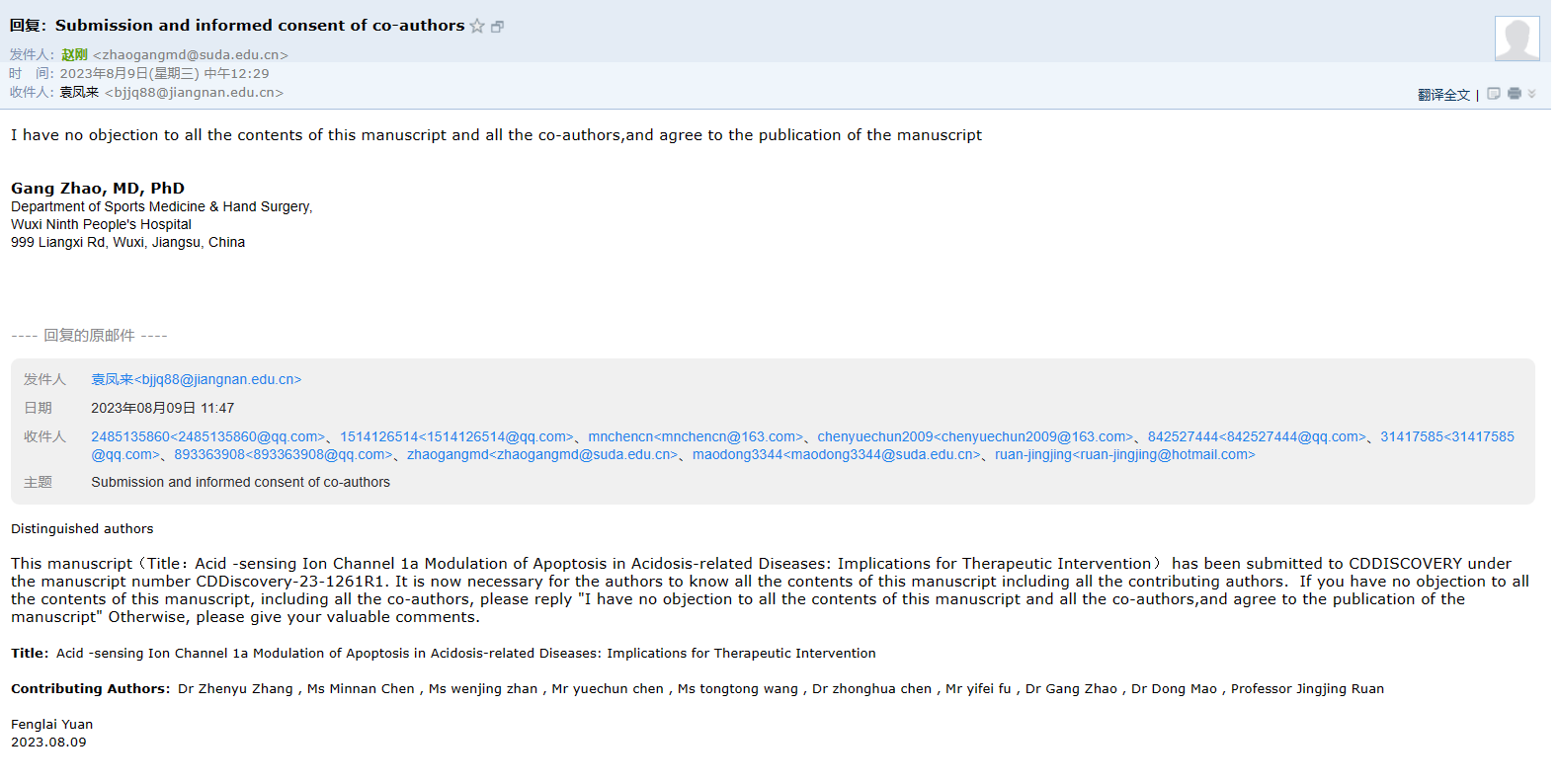

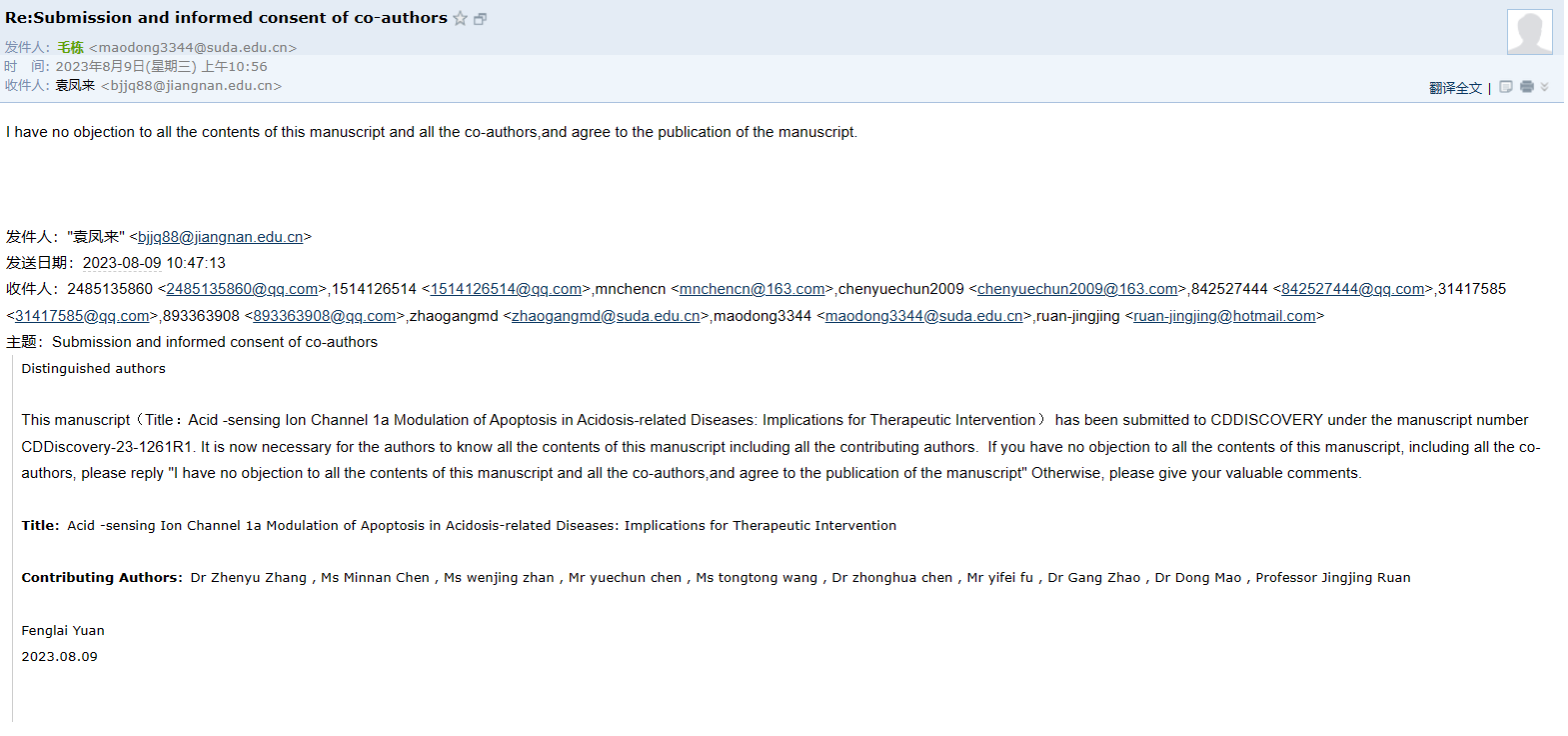

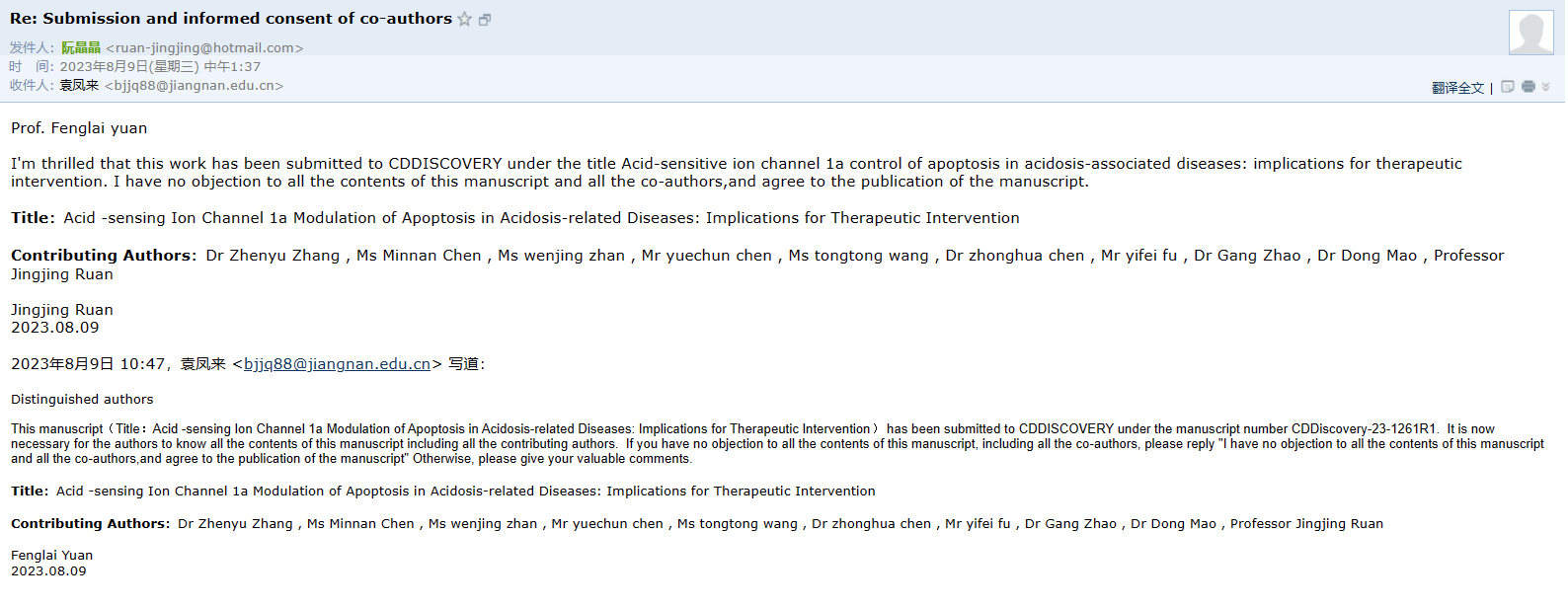

Supplement: Supplementary file 1 — Submission and informed consent of co-authors [file 41420_2023_1624_MOESM1_ESM.docx]
